# Effect of a Novel Intervention Targeting Appetitive Traits on Body Mass Index Among Adults With Overweight or Obesity: A Randomized Clinical Trial

**DOI:** 10.1001/jamanetworkopen.2022.12354

**Published:** 2022-05-18

**Authors:** Kerri N. Boutelle, Dawn M. Eichen, Carol B. Peterson, David R. Strong, Dong-Jin Eastern Kang-Sim, Cheryl L. Rock, Bess H. Marcus

**Affiliations:** 1 Department of Pediatrics, University of California, San Diego, La Jolla,; 2 Herbert Wertheim School of Public Health and Human Longevity Science, University of California, San Diego, La Jolla,; 3 Department of Psychiatry, University of California, San Diego, La Jolla,; 4 Department of Psychiatry and Behavioral Health, University of Minnesota, Minneapolis; 5 Department of Family Medicine, University of California, San Diego, La Jolla,; 6 Behavioral and Social Sciences, Brown University, Providence, Rhode Island

## Abstract

**Importance:**

Behavioral weight loss (BWL) programs result in weight loss for some, but most individuals regain the weight. The behavioral susceptibility theory proposes that genetically determined appetitive traits, such as food responsiveness (FR) and satiety responsiveness (SR), interact with the environment and lead to overeating and weight gain; the regulation of cues (ROC) intervention was developed specifically to target FR and SR.

**Objective:**

To evaluate the efficacy of ROC, ROC combined with BWL (ROC+), BWL, and an active comparator (AC) over 12 months of treatment and 12 months of follow-up.

**Design, Setting, and Participants:**

This randomized clinical trial was conducted from December 2015 to December 2019 in a university clinic. A total of 1488 volunteers from the community inquired about the study; 1217 were excluded or declined to participate. Eligibility criteria included body mass index (BMI) of 25 to 45, age 18 to 65 years, and lack of comorbidities or other exclusionary criteria that would interfere with participation. Data were analyzed from September 2021 to January 2022.

**Interventions:**

ROC uniquely targeted FR and SR. BWL included energy restriction, increasing physical activity, and behavior therapy techniques. ROC+ combined ROC with BWL. AC included mindfulness, social support, and nutrition education.

**Main Outcomes and Measures:**

Change in body weight as measured by BMI.

**Results:**

A total of 271 adults (mean [SD] age, 46.97 [11.80] years; 81.6% female [221 participants]; mean [SD] BMI, 34.59 [5.28]; 61.9% White [167 participants]) were assessed at baseline, midtreatment, posttreatment, and 6-month and 12-month follow-up. Sixty-six participants were randomized to AC, 69 to ROC, 67 to ROC+, and 69 to BWL. Results showed that ROC, ROC+, and BWL interventions resulted in significantly lower BMI at the end of treatment (BMI ROC, −1.18; 95% CI, −2.10 to −0.35; BMI ROC+, −1.56; 95% CI, −2.43 to -0.67; BMI BWL, −1.58; 95% CI, −2.45 to −0.71). Compared with BWL, BMI at the end of treatment was not significantly different from ROC or ROC+ (BMI ROC, 0.40; 95% CI, −0.55 to 1.36; BMI ROC+, 0.03; 95% CI, −0.88 to 0.93); however, the BMI of the AC group was substantially higher (BMI AC, 1.58; 95% CI, 0.72 to 2.45). BMI reductions at 24 months after randomization were similar for ROC, ROC+, and BWL. Importantly, FR was a moderator of treatment effects with more weight loss for participants who scored higher in FR in the ROC and ROC+ groups.

**Conclusions and Relevance:**

These findings suggest that ROC and ROC+ provide alternative weight loss approaches for adults. These models could be particularly effective for individuals who struggle with FR and could be used as a precision approach for weight loss.

**Trial Registration:**

ClinicalTrials.gov Identifier: NCT02516839

## Key Points

**Question:**

What is the effect of regulation of cues (ROC), a weight loss treatment based on appetitive traits, and ROC plus behavioral weight loss (ROC+) compared with BWL alone and an active comparator (AC) among adults?

**Findings:**

In this randomized clinical trial with 271 adults, patients randomized to ROC, ROC+, and BWL demonstrated similar weight loss during treatment and follow-up. Individuals who scored higher on food responsiveness lost more weight in the ROC and ROC+ groups than in the BWL or AC groups.

**Meaning:**

These findings suggest that ROC and ROC+ could be alternative weight loss programs for adults, particularly for individuals with high levels of food responsiveness.

## Teaser

This randomized clinical trial compares the effects of regulation of cues weight loss treatment with behavioral weight loss treatment on body mass index (BMI) in adults with overweight or obesity.

## Introduction

Approximately 74% of US adults are living with either overweight or obesity.^1^ Behavioral weight loss (BWL) programs, or lifestyle intervention programs, are the standard treatments for adults with overweight or obesity. However, trials repeatedly demonstrate moderate weight losses with substantial regain after the intervention ends.^2,3^ In fact, maintenance of weight loss continues to be one of the biggest challenges in the field. An additional complicating factor is that individuals with overweight or obesity are a heterogeneous population, resulting in a wide variability in response to BWL.^4^ This heterogeneity suggests that underlying factors are impacting an individual’s ability to lose weight and maintain weight loss.

The behavioral susceptibility theory (BST)^5,6,7,8^ purports that genetically determined appetitive traits interact with the current food environment, leading to overeating and weight gain. The BST focuses on 2 important aspects of appetite, eating onset driven by food responsiveness (FR) and eating offset driven by satiety responsiveness (SR). Data suggest that both FR^9,10^ and SR^9,11,12,13,14^ are highly heritable and that these durable appetitive traits are shaped by environmental and individual level factors, such as Pavlovian and operant learning, memory, and neural changes related to diet.^15^

We developed the regulation of cues (ROC) intervention, a novel treatment program based on the BST, that targets both FR and SR. ROC uses psychoeducation and experiential learning to promote proactive management of these appetitive traits. Our pilot data suggest that the ROC program is feasible, acceptable, and shows initial positive outcomes among adults who binge eat^16^ as well as children with overweight or obesity.^17,18^

This randomized clinical trial evaluated whether ROC or ROC combined with BWL (ROC+) would improve weight loss across 12 months of treatment and 12 months of follow-up compared with an active comparator (AC) and BWL only. In exploratory analyses, we examined whether FR and SR moderated outcomes.

## Methods

### Design

**Figure 1.**
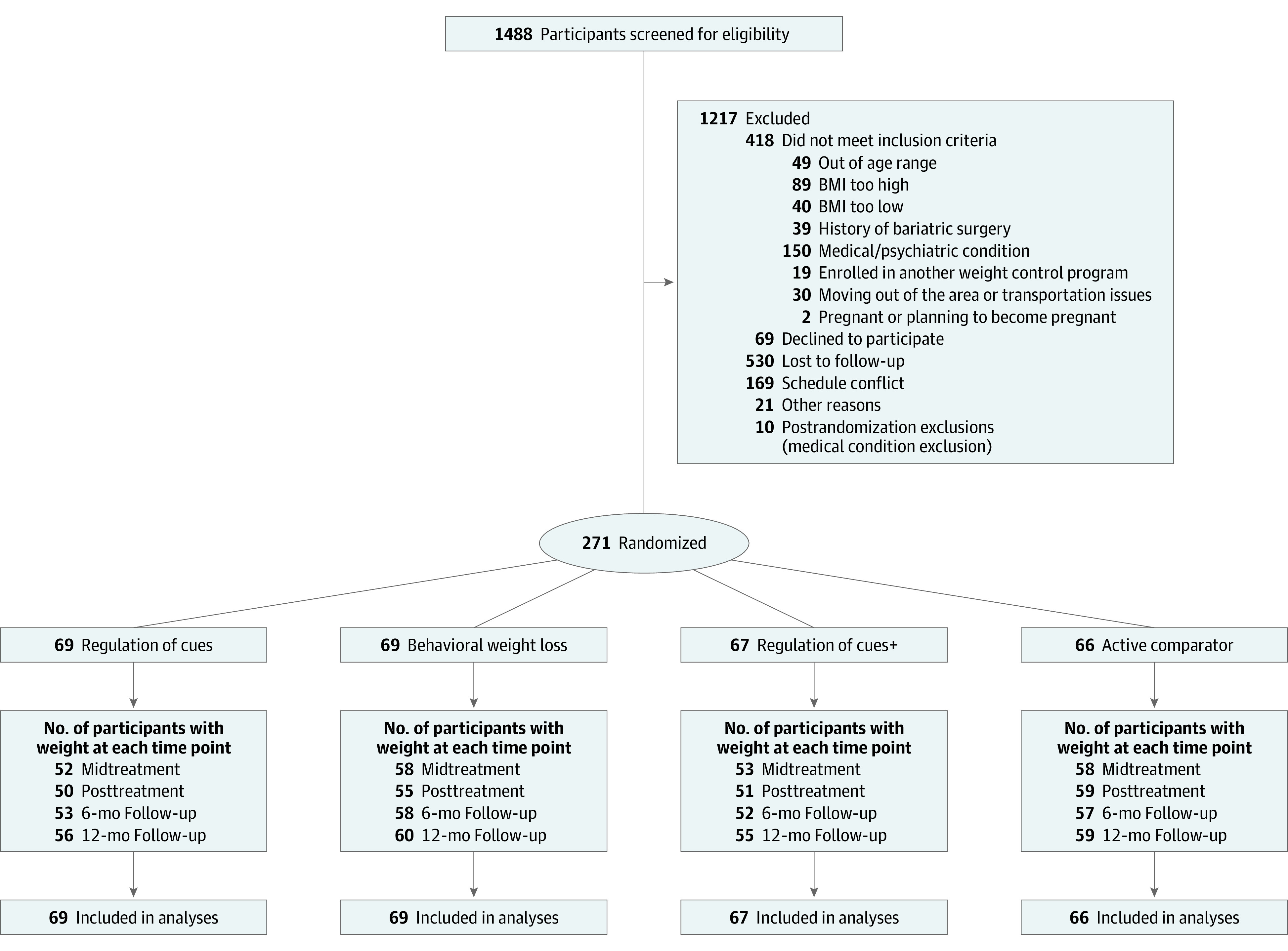
Diagram of Participant Flow Through the Trial

The Providing Adult Collaborative Interventions for Ideal Changes (PACIFIC) study was a randomized clinical trial conducted at the Center for Healthy Eating and Activity Research at the University of California San Diego from December 2015 to December 2019. This report follows the Consolidated Standards of Reporting Trials (CONSORT) reporting guideline for randomized studies (Figure 1). The PACIFIC protocol is available in Supplement 1. In this parallel design, participants were randomly assigned in an equal ratio to 1 of 4 group interventions by the statistician, ROC, BWL, ROC+, or AC using a block randomization procedure conducted with blockrand^19^ with sex and loss of control of eating as blocking factors. Investigators and outcome assessors were blinded to allocations until all assessments were completed. The institutional review board at the University of California San Diego approved the study, and all participants provided written informed consent.

### Participants

Participants were recruited from the San Diego, California, area from December 2015 to November 2017 using radio and online advertisements, ResearchMatch, and referrals from health care practitioners. Adults of any sex and race or ethnicity were eligible if they (1) had a body mass index (BMI; calculated as weight in kilograms divided by height in meters squared) of 25 to 45, (2) were aged 18 to 65 years, and (3) did not meet any other exclusion criteria (eg, type 2 diabetes, recent stroke or angina, pregnancy, inability to speak or read English, or plan to relocate). Detailed inclusion and exclusion criteria can be found in Supplement 1 and have been published.^20^ Empirical power analysis for the trial is published^20^ and supported the planned design of 70 per group, which would provide greater than 0.83 power to detect the planned treatment comparisons with AC (approximately 5% decrease in BMI) with allowance for up to 20% of participants lost to follow-up.

### Intervention

All randomized participants attended 26 90-minute group treatments over 12 months that included 16 weekly sessions, 4 biweekly sessions, and 6 monthly booster sessions. All treatment groups provided the same goal of engaging in at least 150 minutes of moderate or vigorous intensity physical activity per week. Participants were also encouraged to achieve at least 10 000 steps per day. The 3 active treatment groups were provided pedometers and encouraged to self-monitor physical activity.

### Treatment Groups

#### Regulation of Cues

ROC is based on the BST^5,6,7,8^ and included 4 components: psychoeducation to increase awareness of situations, thoughts, moods, and environments that lead to overeating; experiential learning; coping skills; and self-monitoring.^20^ Participants learned about the BST and the ROC targets of improving SR and decreasing FR. Psychoeducation included how physiological responses to food cues develop and can be managed, how the environment encourages the body to overeat, and how to use physiological, behavioral, and cognitive coping skills for mastery and tolerance of FR.

ROC integrated experiential learning to improve responsiveness to hunger or satiety cues (SR) and to decrease sensitivity to food cues (FR). During sessions 2 through 8, participants were taught about hunger and satiety dysregulation. Participants were instructed to self-monitor their hunger on a 1 to 5 scale before, during, and after each meal, either in a self-monitoring booklet or a smartphone application (app). Participants brought dinner to groups and monitored their hunger while simulating different contexts (eg, boredom, sadness, when full, or when hungry).

During sessions 9 through 19, participants learned to self-monitor cravings or urges to eat on a 1 to 5 scale. Starting in session 10, when physically sated, they participated in cue-exposure treatment^17,21,22,23^ and completed 2 exposures with their highly craved foods. Participants self-monitored their cravings outside of the group treatment and tracked the strength, context, how much they ate, and whether they used coping skills. During sessions 20 through 25, participants practiced hunger monitoring during dinner, participated in exposures, reviewed skills, and problem solved issues with implementing the skills.

#### Behavioral Weight Loss

The BWL program recommended a balanced deficit diet based on the US Department of Agriculture’s MyPlate guidelines.^24^ Individualized energy intake goals were based on body weight by multiplying the participant's weight in pounds by 12 to determine an estimate of maintenance energy intake and subtracting 500 and 1000 kcal per day to promote a weight loss of 1 to 2 pounds per week. Behavior change recommendations included stimulus control, self-monitoring, goal setting, managing high-risk situations, meal planning, slowing eating, problem solving, social support, cognitive restructuring relapse prevention skills, and skills for maintaining weight loss. Participants were instructed to self-monitor their food intake, calories, physical activity, and step counts either on paper or using an app.

#### Combined Program (ROC+)

The ROC+ group integrated the focus on diet and energy intake from BWL with the ROC program. Participants learned all of the ROC model components, including psychoeducation, the management of SR and FR, and participated in experiential learning, as well as a focus on decreasing energy intake and behavioral skills from BWL. Participants in this group were taught to self-monitor hunger, cravings, food intake, caloric intake, physical activity, and step counts either on paper or in an app.

#### Active Comparator

The AC program included psychoeducation on diet, stress management, and social support. Participants were provided information about dietary intake and reading food labels. Participants learned about how stress leads to weight gain, as well as mindfulness-based stress reduction, sleep hygiene, and time management. Participants were provided with assertiveness training along with conflict management skills and were encouraged to build positive support networks. At each session, a mindfulness exercise was conducted, and participants were encouraged to practice mindfulness at home. Participants were not instructed to self-monitor.

### Outcome Assessments

Measures occurred at baseline (month 0), midtreatment (month 6), posttreatment (month 12), 6-month follow-up (month 18), and 12-month follow-up (month 24). Participants received the following incentives at assessments: midtreatment, $50; posttreatment, $75; 6-month follow-up, $100; and 12-month follow-up, $175. The primary outcome measure was BMI. FR was measured with the Food Cue Responsivity Scale (FCRS)^25^ and SR was measured with the Reliance on Internal Hunger/Satiety Cues (RHSC) from the Intuitive Eating Scale–2.^26^ Physical activity was measured with the 7-day Physical Activity Recall^27^ and was included as a covariate in all models. Loss of control eating was measured with the Eating Disorder Examination^28,29^ and dichotomized by the presence of any or none reported over the previous 3 months. Body fat was measured using dual-energy x-ray absorptiometry.^30^ Demographics including age, gender, race and ethnicity questions were self-reported. Participants were classified as Hispanic, White, and Non-Hispanic other races (other races included American Indian or Alaska Native, Asian, Black or African American, Native Hawaiian or other Pacific Islander, and multiracial.). Treatment satisfaction and helpfulness was rated with increasing agreement with 6 Likert-scaled items (eg, “I liked the PACIFIC program overall,” and “The skills I learned in PACIFIC were useful”).

### Statistical Analysis

Model parameter estimates were tested using credible intervals (2.5%, 97.5%) and tail probability (2-sided) to asses how likely the value 0 was under the estimated posterior distribution. We used linear mixed-effects (LME) models to compare ROC and ROC+ with AC (aim 1) on changes to BMI, weight (kilograms), and loss of control eating at midtreatment, posttreatment, and 6-month and 12-month follow-up. These models also compared ROC and ROC+ with BWL (aim 2) on posttreatment BMI and rate of change in BMI both during and after treatment (BMI × time). Planned covariates included age, sex, loss of control eating, race or ethnicity, physical activity, and additional corresponding values for BMI or weight at the first session. To examine the robustness of treatment comparisons, we examined temporal patterns of missing BMI measures during and after treatment. Patterns of missed assessment (3 or more consecutive treatment or assessment weights) were classified as (1) no period of missed assessment, (2) missed assessments during the first 6-months of treatment, (3) missed assessments during the second 6-months of treatment, or (4) missed assessments after treatment. Models evaluated dummy-coded contrasts identifying assigned pattern group and whether effects of treatments were moderated by any missing data pattern.

We examined whether treatment effects on weight loss were modified by initial ratings on FCRS and RHSC by adding 2-way interaction terms to primary outcome models. R statistical software version 4.0 (R Project for Statistical Computing)^31^ was used for all analyses, and the lme4^32^ and nlme packages^33^ were used for joint estimation of LME models and imputation models using tools within JointAI^34,35^ for managing missing data. Data were analyzed from September 2021 to January 2022.

## Results

### Participant Characteristics

A total of 1488 volunteers from the community inquired about the study; 1217 were excluded or declined to participate. There were 271 adults randomized, 66 to AC, 69 to ROC, 67 to ROC+ and 69 to BWL. The mean (SD) age of participants was 46.97 (11.80) years, 61.9% (167 participants) were non-Hispanic White, mean (SD) BMI was 34.59 (5.28), and 81.6% (221 participants) were women. Randomization achieved group equivalence in these demographics. Ten additional individuals started treatment but were excluded because of newly reported preexisting medical exclusionary conditions (ie, cancer). Rates of completed postrandomization BMI assessments ranged from 80% to 85% and did not differ across treatments (χ^2^_3_ = 6.4; *P* = .09). During the first 6 months of treatment, 16% of cases had a period of missed assessments; during the second 6 months of treatment, 16% had a period of missed assessments; and after treatment, 13% had a period of missed assessments. Observed patterns of missing assessments did not differ across treatments (χ^2^_9_ = 9.8; *P* = .36). Table 1 lists demographic and initial BMI status for each treatment group.

**Table 1.** Demographics and Baseline Characteristics of the Intention-to-Treat Sample

Characteristic	Participants, No. (%)			
	ROC (n = 69)	BWL (n = 69)	ROC+ (n = 67)	AC (n = 66)
Age, mean (SD), y	46.4 (12.4)	47.6 (11.2)	48.2 (11.1)	45.6 (12.5)
Sex				
Male	12 (17)	13 (19)	13 (19)	12 (18)
Female	57 (83)	56 (81)	54 (81)	54 (82)
Race and ethnicity				
Hispanic	13 (19)	11 (16)	14 (21)	16 (24)
Non-Hispanic other race^a^	15 (22)	12 (17)	11 (16)	11 (17)
White	40 (59)	46 (67)	42 (63)	39 (59)
Body mass index, mean (SD)^b^	35.2 (5.6)	35.4 (5.2)	34.2 (5.6)	34.4 (4.8)
Weight, mean (SD), kg	97.1 (18.3)	97.9 (19.9)	93.8 (17.7)	95.1 (16.1)
Loss of control episodes, mean (SD), No.	0.30 (0.5)	0.30 (0.5)	0.33 (0.5)	0.29 (0.5)

Abbreviations: AC, active comparator; BWL, behavioral weight loss; ROC, regulation of cues; ROC+, regulation of cues plus behavioral weight loss.

^a^ Other racial groups included American Indian or Alaska Native, Asian, Black or African American, Native Hawaiian or other Pacific Islander, and multiracial.

^b^ Body mass index is calculated as weight in kilograms divided by height in meters squared.

### Changes in BMI After Treatment

Mean changes in BMI at the posttreatment assessment indicated weight loss for all 4 groups, AC (mean [SD] −0.32 [2.06]), ROC (mean [SD] −1.29 [2.11]), BWL (mean [SD] −2.55 [2.53]), and ROC+ (mean [SD] −2.03 [2.42]). Likelihood ratio tests of models adding an index of missing pattern group (χ^2^_3_ = 11.9; *P* = .01) and missing pattern group by treatment by time terms (χ^2^_21_ = 33.2; *P* = .04) were each significant. We included the missing pattern group and interactions with treatment in all subsequent intention to treat models.

**Figure 2.**
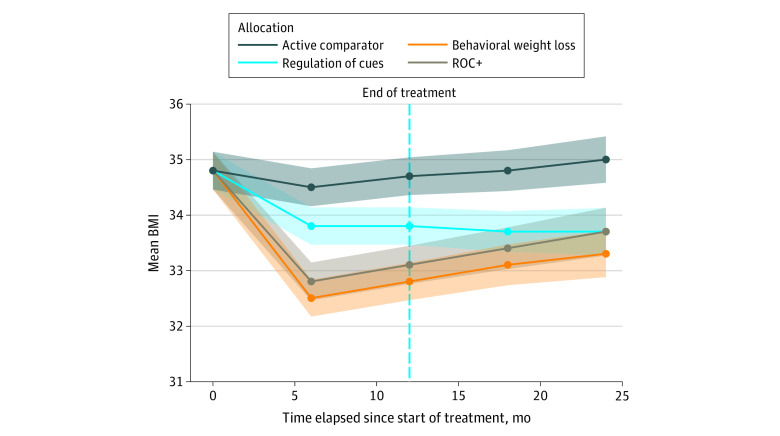
Estimated Marginal Means (SE) for Adult Body Mass Index (BMI) Relative to Assessment at First Session in the PACIFIC Trial ROC+ indicates regulation of cues plus behavioral weight loss.

Joint imputation with 40 replicate combinations were formed using all predictors (age, sex, race or ethnicity, loss of control eating, and physical activity along with adjustment for corresponding baseline on BMI or weight) in the evaluative model. Figure 2 shows the mean changes in BMI from baseline values for participants in each group across all assessments after allocation. Estimates from joint LME models showed that ROC, ROC+ and BWL interventions resulted in significantly lower BMI at the end of treatment (BMI ROC, −1.18; 95% CI, −2.10 to −0.35; BMI ROC+. −1.56; 95% CI, −2.43 to -0.67; BMI BWL, −1.58; 95% CI, −2.45 to −0.71; see Table 2). There were no significant differences in rates of change in BMI after treatment ended for ROC or ROC+ compared with AC. In planned comparisons with BWL, estimates from joint LME models showed that BMI at the end of treatment was not significantly different from ROC or ROC+ (BMI ROC, 0.40; 95% CI, −0.55 to 1.36; BMI ROC+, 0.03; 95% CI, −0.88 to 0.93); however, the BMI of the AC group was significantly higher (BMI AC, 1.58; 95% CI, 0.72 to 2.45). When compared with BWL, participants in AC and ROC+ had similar increases in BMI following the midtreatment assessment and throughout follow-up assessments. Participants in ROC had a significantly different rate of change in BMI through final assessments and did not experience the increase in BMI observed among those in BWL. Evaluation of weight (kilograms) was similar to BMI with significantly lower weight at the posttreatment for ROC, and ROC+ compared with AC and ROC had a significantly different rate of change in weight through final assessments when compared with BWL (Table 2). Results did not support the hypotheses of between-group differences in loss of control eating (Table 2).

**Table 2.** Intention-to-Treat Primary Outcome Analysis of Association Between Treatment Allocation and BMI, Weight, and Loss of Control Eating at Midtreatment (Month 6), Posttreatment (Month 12), 6-Month Follow-up (Month 18), and 12-Month Follow-up (Month 24) Assessments

Intervention^a^	Estimate (95% credible intervals)					
	BMI^b^		Weight, kg		Loss of control eating	
	Comparison with AC	Comparison with BWL	Comparison with AC	Comparison with BWL	Comparison with AC	Comparison with BWL
Difference at the end of treatment						
AC	1 [Reference]	1.58 (0.72 to 2.45)^c^	1 [Reference]	4.43 (2.08 to 6.81)^c^	1 [Reference]	0.10 (−1.34 to 1.52)
ROC	−1.18 (−2.10 to −0.25)^c^	0.40 (−0.55 to 1.36)	−3.29 (−5.78 to −0.80)^c^	1.45 (−1.16 to 4.00)	−0.27 (−1.84 to 1.29)	−0.18 (−1.78 to 1.42)
ROC+	−1.56 (−2.43 to −0.67)^c^	0.03 (−0.88 to 0.93)	−4.79 (−7.21 to −2.39)^c^	−0.44 (−2.97 to 2.09)	−0.10 (−1.07 to 1.79)	0.46 (−1.03 to 1.94)
BWL	−1.58 (−2.45 to −0.71)^c^	1 [Reference]	−4.32 (−6.67 to −1.97)^c^	1 [Reference]	0.36 (−1.54 to 1.33)	1 [Reference]
Difference in rate of change from midtreatment to final follow-up						
AC	1 [Reference]	−0.08 (−0.19 to 0.02)	1 [Reference]	−0.18 (−0.48 to 0.11)	1 [Reference]	−0.02 (−0.13 to 0.10)
ROC	−0.08 (−0.19 to 0.03)	−0.16 (−0.28 to −0.05)^c^	−0.22 (−0.53 to 0.09)	−0.32 (−0.63 to −0.01)^c^	0.07 (−0.05 to 0.19)	0.06 (−0.07 to 0.28)
ROC+	0.05 (−0.06 to 0.16)	−0.03 (−0.14 to 0.08)	0.14 (−0.07 to 0.50)	−0.07 (−0.37 to 0.24)	0.01 (−0.10 to 0.13)	−0.001 (−0.12 to 0.11)
BWL	0.08 (−.02 to 0.19)	1 [Reference]	0.21 (−0.15 to 0.43)	1 [Reference]	0.01 (−0.10 to 0.12)	1 [Reference]

Abbreviations: AC, active comparator; BMI, body mass index; BWL, behavioral weight loss; ROC, regulation of cues; ROC+, regulation of cues plus behavioral weight loss.

^a^ BMI is calculated as weight in kilograms divided by height in meters squared.

^b^ All models include covariates for age, sex, race and ethnicity, physical activity, loss of control eating, type of missing data pattern, and session 1 weight for weight outcome evaluation. Linear mixed effects for time were coded to enable intercept parameters to reflect differences between groups at the end of treatment. Intention-to-treat (ITT) sample model included additional variables to designate missing data group from pattern mixture model. ITT estimates and credible intervals (2.5% to 97.5% intervals reflect a 95% probability of where the true estimate would lie, given the current study data) are estimated from joint imputation models.

^c^ Bayesian tail probability *P* < .05.

Joint LME models of body fat percentage at end-of-treatment and 24-month follow-up with adjustment for planned covariates and baseline levels (eTable in Supplement 2) supported the greatest reductions in mean percentage of body fat at posttreatment in ROC+ (adjusted mean, 43.36%; 95% CI, 41.6%-45.3%) and in BWL (adjusted mean, 43.79%; 95% CI, 41.9%-45.8%) followed by ROC (adjusted mean, 45.48%; 95% CI, 43.2%-47.5%) and AC (adjusted mean, 45.37%; 95% CI, 43.1%-47.6%). We observed an increase in percentage body fat from end-of-treatment to 24-month assessments in BWL (adjusted mean, 45.96%; 95% CI, 44.1%-48.0%) compared with AC (adjusted mean, 46.17%; 95% CI, 44.0%-48.3%).

### FR and SR as Moderators of Treatment Effects on BMI

Modification of treatment effects of ROC, ROC+, and BWL compared with AC on reductions in BMI were examined by baseline levels of FR as assessed by the FCRS and SR as assessed by the RHSC. Exploratory analyses suggested the effect of FR and SR on changes in BMI were not uniform across the range of FCRS and RHSC scores. Restricted cubic splines^36^ enabled evaluation of differences in the association between RHSC scores and FCRS scores and treatment-related changes in BMI. In primary outcome models, sets of terms for 3-way interactions that included change over assessments supported effect modification of treatment-related changes for levels of FCRS and not for RHSC. Participants with higher pretreatment FCRS who received ROC+ sustained greater decreases in weight than participants with high FCRS who received AC (ROC+ vs AC over time interaction with 2 terms capturing the cubic spline at tertiles of FCRS, estimate A (33rd percentile) = 0.009; 95% CI, 0.002 to 0.017; *P* = .03; estimate B (66th percentile) = −0.008; 95% CI, −0.016 to −0.001; *P* = .03).

### Satisfaction With Treatment

On a scale of 1 to 5 (1 = disagree, 5 = agree), on average, participants agreed (mean [SD] score, 4.10 [0.87]) that treatment was helpful, and levels of agreement differed by group (*F*_3,197_ = 11.89; *P* < .001; mean [SD] scores, 4.04 [0.84] for ROC, 4.37 [0.69] for ROC+, 4.27 [0.74] for BWL, and 3.75 [1.02] for AC). Estimated means show ROC+ (mean difference, 0.62; 95% CI, 0.21-1.0) and BWL (mean difference, 0.51; 95% CI, 0.11-0.91) were rated more positively than AC.

## Discussion

To our knowledge, this study was the first randomized clinical trial of weight loss among adults with overweight or obesity participating in the novel ROC intervention. There were no significant differences between ROC, BWL, and ROC+ for weight loss at posttreatment or 12-month follow-up, which is notable given that the ROC group did not include any energy restriction recommendations. Although all treatments lasted 12 months, 3 groups had weight regain (BWL, ROC+, and AC) that started at midtreatment when the visit frequency reduced to monthly. Unlike the other 3 groups, ROC participants stabilized their weight after the midtreatment time point. Importantly, we found that individuals with high levels of FR were more successful in maintaining their weight loss in ROC and ROC+ than those in BWL.

These results are important as ROC is a substantially different treatment than BWL. ROC teaches reliance on internal cues to decrease overeating, whereas BWL focuses on external management skills to decrease overeating. BWL prescribes a diet, restricts energy dense foods, reinforces avoidance of cues to overeat, and focuses on restricting energy intake. ROC does not prescribe a specific diet, trains the use of appetitive cues (instead of energy intake) to guide eating, reinforces tolerance of cravings, and focuses on inhibiting urges to eat palatable food when not physically hungry. Participants in ROC learn to tolerate food cues, not avoid them, and eating is used as a learning experience, which could promote translation of skills outside of the clinic. These skills may be more durable as evidenced by the lack of weight regain in the ROC group. It is important to note that although ROC targets these substantially different processes, there were no significant overall differences between ROC and the other 2 treatment groups that included energy restriction throughout treatment and follow-up.

Dual process theories^37^ suggest that both implicit and explicit processes exert control over behavior. ROC targets implicit processes and teaches internal control of overeating, whereas BWL targets explicit processes and promotes dietary restriction to control overeating. The top-down management of eating, such as in BWL, requires continued effort, which may be depleted over time. These top-down processes also contribute to cognitive load, which when treatment support is reduced or removed, may lead to the reduction of skill use. This may be 1 explanation for why participants in BWL and ROC+ experienced weight regain once visit frequency was reduced. Future studies are needed to further evaluate the impact of weight loss maintenance associated with the ROC treatment.

ROC may confer additional benefits, as participants did not experience weight regain over the 24 months of the study. Weight cycling, including losing and regaining, is associated with depression and poorer mental health^38,39^ as well as disproportionate weight regain.^40^ Additionally, it is possible that participants in ROC could have experienced increased self-efficacy, as they may have been less likely to have experienced feelings of demoralization associated with weight regain.^41^ Further research is also needed to evaluate the effect of ROC on mental health.

Interestingly, we also found that baseline level of FR was associated with improved outcomes in 2 of the 4 treatment groups. Our data suggest that individuals who had higher levels of FR at baseline had more sustained weight loss in ROC and ROC+ than in BWL or AC. This exploratory finding suggests that the appetitive mechanisms targeted in ROC may be especially critical for weight loss among individuals who have trouble resisting food. Thus, ROC could be used as part of a precision approach for weight-loss treatment for individuals with elevated levels of FR.

### Strengths and Limitations

Strengths of the study include the evaluation of a novel intervention, ROC, which is, to our knowledge, the first weight-loss intervention targeting appetitive traits. This study compared ROC, as well as ROC+, with both BWL and an AC to determine the feasibility and initial efficacy. This study included a diverse sample of treatment-seeking adults. Strengths also include the targeting of and measuring specific mechanisms underlying overeating. Physical activity, FR, and SR were assessed using self-report, which may have been influenced by self-report bias. Additionally, we only assessed outcomes using BMI and it is possible that the treatments differentially affected other metabolic indicators. Because it was a treatment-seeking sample, these results cannot be generalized to the general population.

## Conclusions

ROC and ROC+ were successful in facilitating weight loss among adults with overweight or obesity, similar to lifestyle programs such as BWL. The ROC model targets appetitive traits, which is substantially different than BWL and may confer the additional benefit of weight loss stabilization rather than weight regain. This investigation supports the use of ROC and ROC+ as alternative models for treatment of overweight or obesity and could be used in personalized medicine for those individuals with high levels of FR.

## Notes

Supplement 1.
Trial Protocol and Modifications

Supplement 2. **eTable. **Primary Outcome Analysis of Relationship Between Treatment Allocation and Percent Body Fat Measured With Dual-Energy X-Ray Absorptiometry at Posttreatment (Month 12) and 12-Month Follow-Up (Month 24) Assessments

Supplement 3.
Data Sharing Statement
