# Supplementary material for: Effect of a Novel Intervention Targeting Appetitive Traits on Body Mass Index Among Adults With Overweight or Obesity: A Randomized Clinical Trial
Source: JAMA Netw Open. 2022 May 18;5(5):e2212354. doi: 10.1001/jamanetworkopen.2022.12354 (PMC9118075; doi:10.1001/jamanetworkopen.2022.12354)
Supplement: Supplement 1. — Trial Protocol and Modifications [file jamanetwopen-e2212354-s001.pdf]

Title: Effect of a novel intervention targeting appetitive traits on body mass index among adults with overweight or obesity: A randomized clinical trial

Boutelle, K.N., Eichen, D.M., Peterson, C.B., Strong, D.R., Kang-Sim, D.E., Rock, C.L., Marcus, B.

Modifications to the Protocol (excluding staff and advertising changes)

11.6.2015

- Changed name of the study to Providing Adults Collaborative Interventions for Ideal Change (PACIFIC)
- Added pilot study to test the assessment protocol
- Inclusion/exclusion criteria: Removed beta blockers from exclusionary criteria
- Assessment measures: Replaced the International Physical Activity Questionnaire with the Physical Activity Recall interview, and added the Perceived Stress Scale and Food Cravings Questionnaire

1.8.2016

- Inclusion/exclusion criteria: Increased maximum age of enrollment to 65 years
- Assessment measures: Removed the DERS and Emotional Eating Scale and added the Food Responsiveness and Satiety subscales of the Eating Behaviors Questionnaire (EBQ), the Reward-Based Eating Drive (RED) scale, and the Social Support for Diet and Exercise questionnaires
- Incentives: Increased to the following: mid-treatment $50, post-treatment $100, mid-follow-up $100, follow-up $200. For participants who only will provide weight at posttreatment and/or follow-up periods, a compensation of $25 is offered.
- Increased potential number of participants per group to 20 participants

5.24.2016

- Inclusion/exclusion criteria: Added the following exclusionary criteria; bariatric surgery, other members of the same household participating in the study, and participation in another weight control program
- Assessment measures: Added the Self-Compassion Scale and Control of Eating Questionnaire

11.4.2016

- Addition of an ancillary study to develop an episodic memory task for food cues which utilizes baseline data from the parent study and adds a healthy weight comparison group
- Assessment measures: Removed DXA scan and added the following measures: a modified version of the California Verbal Learning Task (CVLT) that includes food words, digit span, a food specific directed forgetting task, and the CVLT-II (for the healthy weight comparison group only)
- Incentives: Due to removal of the DEXA scan, assessment compensation was modified to the following: mid-treatment $50, post-treatment $75, mid-follow-up $100, follow-up $175.

8.8.2017

- Assessment measures: Added the CVLT-Food to all follow-up assessments

UCSD Human Research Protections Program
New Biomedical Application
RESEARCH PLAN

Instructions for completing the Research Plan are available on the HRPP website.
The headings on this set of instructions correspond to the headings of the Research Plan.
General Instructions: Enter a response for all topic headings.
Enter "Not Applicable" rather than leaving an item blank if the item does not apply to this project.

Version date: 9/30/2013

1. PROJECT TITLE

Treatment of obesity targeting appetite and cue reactivity

2. PRINCIPAL INVESTIGATOR

Kerri Boutelle, Ph.D
David Strong, PhD, Co-I
Cheryl Rock, PhD, Co-I
Bess Marcus, PhD. Co-I

3. FACILITIES

UC San Diego
La Jolla Village Professional Center
8950 Villa La Jolla Drive, Suite C-203
La Jolla, CA 92037

4. ESTIMATED DURATION OF THE STUDY

5 years

5. LAY LANGUAGE SUMMARY OR SYNOPSIS (no more than one paragraph)

This study tests the efficacy of the Regulation of Cues (ROC) treatment and ROC + Behavioral Weight Loss (BWL) compared to an attention control (AC) and to BWL. Treatment will be delivered over 1 year and we will follow up with participants 1 year post-treatment. 280 overweight and obese adults who binge eat will be randomized into one of four treatment arms. It is estimated that 400 individuals will be recruited to achieve this goal. In the ROC treatment, participants will be trained to increase awareness of hunger and fullness signals and avoid eating in response to cravings, through the use of a variety of skills taught in group. The four arm design will allow us to compare the Regulation of Cues treatment (ROC) as a stand-alone treatment and ROC+Behavioral Weight Loss to both an active control and the gold standard BWL.

6. SPECIFIC AIMS

The primary aim is to compare ROC and ROC+BWL to AC, and to BWL, on body mass index (BMI), %weight, %body fat, and binge eating over the course of treatment and follow-up. The secondary aim is to compare ROC, ROC+BWL, BWL and AC on sensitivity to appetitive cues, sensitivity to food cues, inhibition, restriction, caloric intake, overeating, and physical activity over the course of the treatment and follow-up.

7. BACKGROUND AND SIGNIFICANCE

Obesity has become a national epidemic with significant implications in physical and psychological functioning, increasing healthcare costs, and high mortality rates. Binge eating is a common maladaptive eating behavior observed in obese samples. About 10 to 15 percent of people who are mildly obese have binge eating disorder. Prevalence rates of binge eating are about 10-15 percent in mildly obese samples, and substantially higher in samples of severely obese people. Binge is associated with higher weight status, body image concerns, depression, and even poorer weight loss following bariatric surgery. Despite the high prevalence rates and comorbidities associated in this population, the majority of weight loss treatments for obese adults focus very little on the reduction of binge eating and eating in the absence of hunger, yielding these treatments less effective for people who engage in these behaviors.

One treatment study developed by Dr. Boutelle and her colleagues suggests binge eating often results from hyper responsiveness to food cues in the environment. Based upon principles of behavioral psychology proven effective for

conditions such as depression, anxiety, and bulimia, an exposure-based treatment protocol can potentially help patients to habituate to food cues, resulting in reduced binge eating and improved self-efficacy to controlling eating behavior.

Dr. Boutelle has recently implemented her food cue sensitivity program in group-based treatment among an adolescent population, with the treatment showing a statistically significant reduction in binge eating and reduced eating in the absence of hunger. These reductions remained reliable at 6-month and 12-month time points, suggesting that intervention effects could create stable changes in binge eating and emotional eating characteristics.

A logical next step to Dr. Boutelle's food cue sensitivity program is to validate the protocol within adult binge eating populations. The premise of this proposed project is to test this intervention separately with adult participants, by comparing ROC and ROC+BWL to AC, and to BWL.

8. PROGRESS REPORT

This is a new application.

9. RESEARCH DESIGN AND METHODS

Research Design and Methods

Study Overview. This proposal is a randomized controlled study with four arms: ROC, ROC+BWL, BWL and attention control (AC). The ROC program provides psychoeducation (ways the environment "tricks" the body into overeating past nutritional needs, coping skills designed to assist in mastery and toleration of food cue sensitivity, daily self-monitoring (hunger, satiety, and cravings) and experiential learning (exposure sessions while self-monitoring hunger, satiety, and cravings). In the ROC+BWL arm, participants will be taught to decrease caloric intake and increase physical activity, in addition to experiential learning exercises, and psychoeducation. The BWL program will include dietary recommendations, physical activity recommendations, and behavioral change recommendations. The AC arm will be matched to the other groups on number of meetings and incentives; it will consist of psychoeducation including topics on nutrition (multiple sessions), healthy cooking, reading food labels, avoiding the sun, time management, physical activity, lifestyle exercise, eating out, assertiveness skills, coordinating your medical treatment, relaxation training, shopping on a budget, caffeine and alcohol, sedentary behavior, and stress management. (See Table 1)

Table 1. Key components and differences between the four arms.

	ROC	BWL	ROC+BWL	AC
**Dietary prescription**	No dietary prescription. Sessions focus on learning to control physiological and psychological responding to food, and to eat less of foods that are palatable. No education about portion control or food labels.	Restrict calories to 1200 or more based on weight using a low fat low calorie diet. Sessions include problem solving barriers to following the diet, learning about food labels, food shopping, cooking and portion control.	Restrict calories to 1200 or 1500 based on weight using a low fat low calorie diet. Participants learn to control physiological and psychological responding to food, as well as food labels, shopping, cooking and portion control	Healthy eating using choosemyplate.gov. Participants learn about food labels, shopping, cooking and portion control.
**Self-monitoring**	Hunger and craving	Food intake	Food intake, hunger and craving	None
**Experiential learning**	Participants bring meals and/or palatable foods to each session. Hunger and satiety is monitored in session during meals. Exposures to highly craved foods are conducted in session.	None	Participants bring meals and/or palatable foods to each session. Hunger and satiety is monitored in session during meals. Exposures to highly craved foods are conducted in session.	None
**Physical activity prescription**	Physical activity is used to help regulate physiological and psychological responding to food cues. Actual program is the same as BWL.	Physical activity is used to burn calories to assist in weight loss.	Physical activity is used to burn calories to assist in weight loss, as well as to help regulate responding to food cues. Actual program is the same as BWL.	Physical activity is recommended for health. Walking is recommended.
**Stimulus control**	Focus is on toleration and mastery of physiological/ psychological arousal at restaurants and parties. Does not recommend avoiding any eating situations.	Recommend removing palatable energy dense foods from the home, and planning ahead for eating in restaurants and parties, and avoiding high risk eating situations.	Toleration and master of physiological and psychological arousal to food cues, as well as removing palatable energy dense foods from the home, and minimizing and preparing for high risk situations.	None
**Goal setting**	Goal setting focuses on self-monitoring and practicing mastery and toleration of physiological /psychological arousal.	Goal setting focuses on self-monitoring, and adherence to diet and physical activity.	Goal setting focuses on self-monitoring (food intake, hunger and cravings) and adherence to diet and physical activity	None
**Coping skills**	Methods for managing psychological and physiological arousal. Discussed each week in regards to mastery and toleration of physiological /psychological arousal.	Discussed in terms of how to cope to reduce barriers to diet and physical activity adherence	Methods for managing psychological and physiological arousal. Discussed each week in regards to mastery and toleration of physiological /psychological arousal.	None
**Other health issues**	None	None	None	Sleep, seat belt use, decreasing alcohol consumption, use of sunscreen. Etc.

We will recruit 400 adults to randomize 280 overweight and obese adults meeting all inclusion criteria, will provide 1 year of treatment and will follow participants 1 year post-treatment. We will conduct 5 assessments; baseline (2 visits to CHEAR; 1 visit to ePARC), mid-treatment (month 6), post-treatment (month 12), mid follow-up (month 18) and follow-up (month 24). Participants may be asked to complete interviews and self-report measures, such as those listed below. Assessments will include measures of anthropometry, nutritional intake, physical activity, responsivity to internal hunger/satiety cues, responsivity to food cues, eating behavior and cognitions. We will evaluate potential mediators of effect during treatment, including bi-monthly assessments of appetite awareness, food cue responsivity, and perceptions of restriction of foods. We will also collect data on acceptability, liking, self-monitoring frequency, number of sessions attended and retention.

Research Design. This study will randomize 280 overweight and obese adults by binge eating level and gender to one of four arms (ROC, ROC+BWL, BWL and AC). The study data manager, who will have no contact with research participants, will conduct randomizations and monitor allocations throughout the study to alert the PI of any systematic differences in additional demographics (age, race/ethnicity) that may arise.

Quality control, fidelity and supervision of intervention. The intervention for this study will be provided by trained postdoctoral fellows and licensed clinical psychologists. Close monitoring through multiple methods will be used to ensure treatment integrity. All ROC group leaders will attend a 2-day training with Dr. Boutelle ,Dr. Neul, and , Dr. Eichen, and will meet weekly with Dr. Neul for treatment supervision. BWL and AC group leaders will attend a 2-day training with Dr. Rock and will meet weekly with Dr. Rock for treatment supervision. Intervention sessions will be audiotaped for ongoing performance monitoring. Random samples (e.g. 30% or more) of all intervention sessions will be rated for fidelity by an independent rater using a measure created for this study. These supervision and monitoring processes have been successful in our previous treatment studies, in our current projects (R01 DK075861; R01KD094475) and we expect that they will assure treatment fidelity in this project.

Quality control, fidelity and supervision of assessments. All assessors will be advanced graduate students, postdoctoral fellows or clinical psychologists who will attend a 2-day training with Dr. Peterson and Dr. Liang. All assessors will be videotaped and scored for adherence to the assessment procedures and certified by Dr. Peterson and Dr. Liang before working with study participants. All assessors will meet weekly with Dr. Liang and Dr. Peterson (as needed) for assessment supervision. Assessment sessions will be audiotaped for ongoing performance monitoring. Random samples (e.g. 30% or more) of all interview sessions will be rated for fidelity by an independent rater using a measure created for this study. Dr. Liang has developed protocols for the psychophysiological assessments for our other project (R01KD094475). Assessment staff will be blind to treatment condition.

***Remuneration and Maximizing Retention*.** All participants will receive incentives in the form of gift cards at the following levels for time and effort: mid-treatment = $25, post-treatment = $50, mid-follow-up = $25, and follow-up = $100. A systematic protocol will be followed to minimize subject attrition. Participants who miss a visit will be called by the group leader for counseling by telephone unless the missed visit was reported in advance (e.g., vacation). For each data collection visit, participants will be scheduled by telephone, sent written reminders, and called the day before the visit. Missed data collection visits will be rescheduled and followed up at least 3 times. If necessary, transportation to the clinic will be provided.

In order to ensure high retention of our sample, we will request personal e-mail addresses and cell phone numbers as well as contact information for two close friends or relatives to further enhance our ability to locate participants. In addition, after the first data collection is complete, we plan to send winter and summer holiday cards and newsletters in order to maintain updated addresses that will permit future contacts. Even if the participant moves multiple times before the follow-up survey administration, interim address information will be helpful in confirming location of an individual.

Treatment Arms

All interventions arms will be matched on contact frequency and time. All treatment groups will be 1.5 hours (including weigh-ins) and will be provided in groups of 10-15 participants weekly for 4 months, twice a month for 2 months and monthly for 6 months (total treatment duration = 12 months, 26 meetings). Key differences between the treatment arms are outlined in Table 1.

Regulation of Cues (ROC). The ROC program provides psychoeducation, coping skills, self-monitoring and experiential learning.

Psychoeducation. The ROC program provides psychoeducation at each group visit by describing a "Deceptive Hunger", which is a way that the environment "tricks" the body into overeating past nutritional needs. The overall goal of psychoeducation is to increase participant's awareness of the reasons why they may overeat, and to relieve participants from guilt regarding overeating by helping them understanding the processes by which these phenomena occur. Both lack of sensitivity to appetite and satiety cues and increased sensitivity to food cues will be discussed. Physiological, neurobiological and environmental models of overeating past nutritional needs are presented in lay language so that participants can understand their vulnerabilities to overeating. The concepts are taught using a chronic disease model in which the individual is considered to have the biological vulnerability to overeat that is amplified by the current obesogenic food environment. Participants are provided information about basic learning theory and how physiological responses to food cues develop and can be managed.

Coping skills. Coping skills will be taught to accompany each Deceptive Hunger. Coping skills are presented to assist in mastery and toleration of food cue sensitivity. Coping skills include physiological skills (deep breathing, relaxation, and mindfulness), behavioral skills (delay, activity substitution) and cognitive skills (cognitive restructuring, distraction).

Experiential learning and self-monitoring. In each session, participants will complete an experiential learning exercise. During visits 1-8, participants will be taught about hunger and satiety dysregulation. Participants will be taught to monitor their hunger in a self-monitoring booklet on a 1-5 scale, with 1 "starving" and 5= "stuffed". Participants will be instructed to self-monitor hunger and satiety before, during and after each meal, as well as 10 and 20 minutes after eating for a minimum of two meals/snacks per day. Participants will bring dinner and all groups will start by eating dinner and monitoring their hunger with prompting from the group leader.

During visits 9-16, participants will learn to assess and rate their cravings (defined as urges to eat when not physically hungry). Craving is monitored with a 5-point scale, 1= "not craving it at all" and 5="craving is overwhelming" and participants will rate cravings during the day (ideally one craving a day at minimum). Participants will create a craving hierarchy and will bring their highly craved foods to group and will complete an exposure at each session (CET-Food). Exposures are only conducted when participants are not physically hungry. If a participant is physically hungry, they will have a snack before participating in an exposure. During the exposure, participants will rate their cravings while looking at the food, holding the food, smelling the food, after taking two small bites of the food, and then will rate their cravings at 30-second intervals for the duration of the exposure. After 10 minutes, the participants dispose of the food without eating it and the exposure ends.

In all the following weeks participants will monitor both their hunger and cravings.

Physical activity. For this version of ROC, we will prescribe the same physical activity program as BWL; however, the integration of physical activity will be promoted to improve self-regulatory strength and to help participants master and tolerate physiological and psychological arousal, resist cravings and overeating.

Behavioral Weight Loss (BWL). The BWL program will include dietary recommendations, physical activity recommendations, and behavioral change recommendations.

Dietary recommendations. All participants will be instructed on how to consume a balanced deficit diet of conventional foods that provide ~15–20% of energy from protein, 30% or less energy from fat, and the remainder from carbohydrate. Individual goals for energy intake will be based on initial body weight. Participants who weigh ≤249 lb will be prescribed 1,200 kcal/day, whereas those 250–299 lb will be prescribed 1,500 kcal/day, with higher allotments for heavier individuals (i.e., 300–349 lb, 1,800 kcal/day; ≥350 lb, 2,000 kcal/day). Participants will be instructed in measuring portion sizes, counting calories (with a calorie counter provided or on their phone), and self-monitoring food intake.

Physical activity recommendations. The program will focus on increasing both lifestyle activity and structured exercise programs. The physical activity program will include a structured exercise goal progressing initially to 250 min/week. The long-term goal will be an average of at least 60 min/day of purposeful exercise at a moderate level of intensity, which is consistent with current recommendations for weight management.88 Lifestyle activity goals focus on building increased activity into typical, daily activities, such as walking or bicycling. Participants are also instructed to decrease sedentary behaviors, such as TV watching or computer usage outside of work. Participants will be given a pedometer and will be encouraged to work toward achieving 10,000 steps per day. In all aspects of increased physical activity, standard behavioral elements such as convenience, enjoyment, time management, managing the environment, and social support will be addressed.

Behavior change recommendations. Behavior change recommendations include stimulus control, self-monitoring, goal setting, managing high-risk situations, meal planning, slowing eating, problem solving, social support, cognitive restructuring, lapse and relapse prevention skills, and maintaining weight loss.

BWL+ROC. BWL and ROC will be integrated for this arm, to capitalize on the strengths of both treatments. All participants will be taught to decrease caloric intake and increase physical activity, and to use all of the behavioral skills provided in BWL. However, they will also be taught models of hunger and satiety and about food cue reactivity, and will learn skills to manage these. This arm will include an experiential component, including hunger monitoring during dinner and participating in CET-Food in the clinic.

Active Control (AC). In order to equate for contact time received by participants in the other three intervention arms, the AC will be matched on number of meetings and incentive components. Topics included will be sleep hygiene, nutrition (multiple sessions), healthy cooking, shopping on a budget, reading food labels, physical activity, avoiding the sun, time management, eating out, assertiveness skills, coordinating your medical treatment, relaxation training, mindfulness training, caffeine and alcohol, sedentary behavior, lifestyle exercise, and stress management. Even though nutrition and physical activity will be included, no information will be provided regarding making behavioral changes toward these goals. These types of active controls have been very successful in our other studies and have had high retention rates.

Measurements (Nonstandard measures will be described in the text, published measures in Table 2)

Anthropometry Height will be measured using a portable Schorr height board (Schorr Inc, Olney, MD) in duplicate. Height will be recorded to the nearest 0.1 cm for both trials, and the average of the 2 values will be used for analysis. Body weight in kilograms will be measured in duplicate on a Tanita Digital Scale (model WB-110A). Body weight will be recorded to the nearest 0.1 kg and the average of the 2 values will be used for analysis. Height and weight will be converted to body mass index (BMI=[kg/m2]).

Additionally, fat mass, fat free mass, resting metabolic rate and total energy expenditure will be measured using the FDA approved dual x-ray absorptiometry, DXA. This appointment will take place at the Exercise and Physical Activity Resource Center (ePARC).

Psychophysiological Measurements. All of the psychophysiological measures were chosen as measures of Cephalic Phase Responses (biological preparatory responses to food) and have shown sensitivity to conditioning paradigms with food.1,2,3Cephalic Phase Responses (CPRs) have been associated with responses to food and perceived craving in humans1 and differentiate overweight and normal weight adults.4 Electrophysiological recordings will all be sampled at 250 Hz. Participants will be exposed to highly palatable food for 6 minutes and a neutral object for 2 minutes.

Salivation. Salivation will be measured using swallows recorded by electromyography.5The accompanying EMG signal will be recorded with three Ag±AgCl electrodes, two electrodes will be attached under the left jaw, in the length of the anterior part of the musculus digastricus and a reference electrode will be placed on the left mastoid process. The definition of a swallowing response will be determined as a response of the integrated signal above a 5 mV threshold.5

Heart rate and heart rate variability. Heart rate and heart rate variability will be measured using two Ag±AgCl electrodes, one attached on the left side of the subject, the other attached under the right collarbone. 6 R-waves will be detected off-line with a template matching procedure, and inter-beat intervals will be calculated. Heart rate variability (HRV) represents the continuous interplay between the parasympathetic and sympathetic influences on heart rate, and measures the capacity for regulated responding. Frequency based

HRV analyses are based upon the variations in heart rate modulated by the sympathetic nervous system and parasympathetic nervous system.7 Three main spectral components can be calculated based on the HRV measurements: very low frequency (VLF), low frequency (LF), and high frequency (HF). The LF/HF ratio is considered to reflect the balance between parasympathetic and sympathetic autonomic activity. Heart rate (HR) and heart rate variability (HRV) will be measured continuously during the food exposure tasks.8

Skin conductance. Skin conductance will be measured with Biopac System (Biopack Systems, Inc). Two Ag±AgCl electrodes will be placed on the thenar and hypothenar eminences of the palm of the non-dominant hand.

Stop signal task with food pictures. The stop signal task is designed to measure response inhibition in a laboratory paradigm. This study will utilize a food version of the traditional stop signal task. In this version, there are two concurrent tasks: a go task, which is a choice reaction time task, and a stop task, which involves inhibiting responses to the go task. Go stimuli are four pictures of food in landscape or portrait format; participants must respond to the go stimuli by pressing the left and right response keys on the keyboard (left for portrait and right for landscape). On 25% of trials, a visual stop signal will be presented; participants are instructed to withhold responding when this signal is presented. Higher stop signal reaction times indicate decreased inhibitory control.

Visual probe task. The visual probe task is based on the task used by Brignell and colleagues to assess attentional bias to food cues in adults, and it is similar to the task used by Bradley and colleagues to measure attentional bias to aversive and appetitive stimuli. Each trial commences with a fixation cross, which is displayed for 500 ms in the center of the screen. Attentional bias will be measured using response times (RTs) to probes replacing pictures of food and not the control cue.

Stroop (Food). The original Stroop task is a well-established measure of cognitive interference that assesses information processing biases. The food modified Stroop measures differences in reaction times to naming the color of food-related versus neutral words. Slower naming of food words are presumed to measure attentional biases for food, because attention toward food words may interfere with the primary color-naming task. The present study will use a

computer modified food version of the Stroop task.

Treatment Acceptability and Liking. This survey will assess ease of use, acceptability, and perceived usefulness of the intervention.

Data collection protocol. The data for this study will include a number of different methods of collecting data. The survey will be available as a secure web-based survey. The psychophysiological data will need to be post-processed and entered, and will be duplicate entered to minimize error and supervised by Dr. Liang and the data manager. The data manager will evaluate range and means of all data collected, and will identify data entry errors weekly. The data manager will create scales, and will verify range and means. The data manager, along with Dr. Strong, will merge the data in preparation for analyses. These protocols have been successful in our other studies.

ANALYSIS PLAN

Preliminary analyses. We will begin with an examination of the distribution of key variables to assess their characteristics, to provide descriptive statistics of the study population, and to allow assessment of randomization. Outliers will be identified and variables whose distributions depart significantly from normality will be evaluated for alternate estimators using generalized LME models. Psychometrics of self-reports will be examined to determine scale reliability and test information.

Primary outcome will include comparison of ROC and ROC+BWL, with AC interventions on changes on BMI, weight (%), body fat %, and binge eating at 6-, 12-, 18- and 24-month assessment after baseline (PA1). Additional primary outcomes compare ROC and ROC+BWL to BWLon changes on BMI, weight (%), body fat %, and binge eating at 6-, 12-, 18- and 24-month assessment after baseline (PA2). Secondary aim will evaluate planned treatment group comparisons on changes in sensitivity to appetitive cues, sensitivity to food cues, inhibition, restriction, caloric intake, overeating, and physical activity (total physical activity and moderate/vigorous minutes/week) (SA1). All other study endpoints including behavioral and psychological outcomes are considered secondary or exploratory.

Primary outcome analysis (PA1 and PA2). We will evaluate the main effects of treatment allocation on change in BMI, %weight lost, body fat %, binge eating across baseline, 6-,12-,18-, and 24 months. LME models with dummy-coded indicators for planned treatment comparisons (ROC and ROC+BWL vs AC, ROC and ROC+BWL vs BWL; ROC+BWL vs ROC and BWL) and a continuous term for time selected after evaluating non-linearity. Planned covariates will include gender, baseline binge eating status, and baseline values for assessing corresponding primary outcomes (PA1, PA2). The LME model provides maximum likelihood parameter estimates based on all of the available data, allowing for the inclusion of cases with missing data and the modeling of the covariance error structure of the data across the assessment points.

	Instrument (references)	Time-point					
		**1**	**Trt visits**	**2**	**3**	**4**	**5**
**Demographics**	Age, gender, ethnicity, income	X					
	Barratt Simplified Measure of Social Status	X					
**Anthropometry**	Height and Weight (BMI)	X	X	X	X	X	X
	Body composition (DXA)	X			X		X
**Psychological and medical disorders**	Eating Disorder Evaluation (Binge eating, eating disorders)	X		X	X	X	X
	Binge Eating Scale	X	X	X	X	X	X
	EDEQ	X	X	X	X	X	X
	PHQ-9	X		X	X	X	X
	GAD-7	X		X	X	X	X

	DERS	X		X	X	X	X
	UPPS-P	X		X	X	X	X
	Executive functioning Index	X		X	X	X	X
	MINI	X					
	SCID II –BPD	X					
	Medication/medical history questions	X	X	X	X	X	X
**Sensitivity to appetitive cues**	Intuitive Eating Scale [62]	X	X	X	X	X	X
**Sensitivity to external Food cues,**	Psychophysiological measurements	X			X		X
	Power of Food Scale [103]	X		X	X	X	X
	Visual Probe Test	X		X	X	X	X
	Stop Task with food pictures	X		X	X	X	X
**Inhibition to food cues**	Food stroop	X		X	X	X	X
**Eating behaviors, intake, and related cognitions**	Dietary questionnaire	X		X	X	X	X
	Eating in the absence of hunger questionnaire	X	X	X	X	X	X
	Emotional Eating Scale	X		X	X	X	X
	Three Factor Eating Questionnaire (Restraint)	X	X	X	X	X	X
**Physical Activity**	GODIN Leisure-time exercise questionnaire	X		X	X	X	X
	iPAQ	X		X	X	X	X
**Treatment Acceptability**	Treatment Acceptability and Liking				X		

10. HUMAN SUBJECTS

Overall, we expect to consent 400 individuals to randomize 280 adults who meet the following eligibility criteria:
Enrollment criteria:

1. All participants will be between the ages of 18-55 meeting criteria for overweight, with a BMI between 25 and 40.
2. Participants will provide written informed consent for study participation.
3. Participants will possess English language skills at the 5th grade reading level.
4. Participants will be free of major medical conditions such as a recent history of coronary heart disease; recent history of myocardial infarction; recent symptoms of angina, diabetes, recent stroke, orthopedic problems that would limit activity during the following twelve months; or any other serious medical condition that would make physical activity unsafe.
5. Participants will not have bulimia or anorexia, significant cognitive impairment, a known psychotic disorder, or unstable psychiatric illness (e.g., recent psychiatric hospitalization, acute suicidal ideation) as derived from their intake interview and questionnaires.
6. Participants will not be moving out of the San Diego area for the duration of their study enrollment (24 months).
7. Participants will not be pregnant, planning to get pregnant in the 2 year study period or lactating.
8. Participants will not be taking medication for weight loss or that may impair physical activity tolerance or performance (e.g., beta blockers).
9. Participants with medical or psychological problems, or taking medications that could make adherence with the study protocol difficult or dangerous will not be included.

11. RECRUITMENT AND PROCEDURES PREPARATORY TO RESEARCH

Participants will be recruited from the San Diego Metropolitan area using online advertisements such as Craigslist, flyers to physicians, flyers posting on campus and direct mailings and direct email to participants, radio ads, ResearchMatch,

and professional referrals to the lab from local physicians. If needed, information for direct mailing/emailing will be purchased from companies or services such as the U.S. Postal Service that allow you to purchase/ pay to send mail or email to individuals. Participants who respond to recruitment efforts will be asked to complete an initial online screen to determine initial eligibility if individuals do not have online access, they can complete an extended screening call that will include these questions as part of the telephone screen. Participants who meet study inclusion criteria will then complete a phone screen to further assess eligibility. If participants meet initial screening criteria, they will be scheduled for an orientation to learn more about the study, review the informed consent and have all questions answered. If they remain interested in participating, they will then sign an informed consent and be scheduled for the first assessment to determine eligibility.

12. INFORMED CONSENT

The online screening will assess for basic inclusion and exclusionary criteria. This process presents no more than minimal risk of harm to subjects, and involves no procedures, for which written consent is normally required outside of the research context. For these reasons, we request a waiver of documented consent for the online screening. For individuals who do not have online access, the questions asked on the online screen can be administered over the phone in conjunction with the phone screen.
The phone screening will assess for basic inclusion and exclusionary criteria. This process presents no more than minimal risk of harm to participants and involves no procedures for which written consent is normally required outside of the research context. For these reasons, we request a waiver of documented consent for the phone screen.

All participants will complete written informed consent prior to enrollment at an orientation session. It is not likely that participants for this population will lack the capacity needed for consent. If there are any concerns, to ensure participants understand the consent form, they will be asked to describe what the study is about. For participants in which capacity for consent is judged to be questionable, they will complete the Mini-mental State Examination (MMSE) and if they receive under a 24, they will not be eligible for enrollment (i.e., surrogate consent will not be acceptable). It is highly unlikely that the MMSE will need to be administered to this patient population but if it is administered, documentation of the test results will be kept in the participant file.

The orientation and consent process may take place with a group of potential participants. Participants may ask questions as a group and they will also be informed that they can each have an opportunity to privately ask additional questions. If individuals are unsure as to whether they are willing to participate in the study, they may take the consent form home with them. If they later decide they wish to participate, they may schedule their first assessment and sign the consent form prior to participating in the assessment.
CHEAR staff members able to provide information about the study and carry out the consent procedures include Kerri Boutelle, June Liang, Dawn Eichen, Teresa Monreal, Ashley Ryan, Brittany Matheson, Zoe Mestre

13. ALTERNATIVES TO STUDY PARTICIPATION

The alternatives to participation in this study are to not participate and to seek treatment with another therapist or community program.

14. POTENTIAL RISKS

1). Potential risk of psychological assessments. For some participants, disclosing potential information about mental health symptoms and eating behaviors may be uncomfortable. Questions regarding individual behaviors, emotions or attitudes may be considered sensitive to some participants.

2). Potential risk of psychological treatment. The active treatments will involve exposure to food while restricting consumption of said food. This may provoke negative emotions for some participants.

3). Potential risk of loss of confidentiality: Risk associated with breach of confidentiality of behavioral research data. Since this study includes psychological assessments as well as height and weight, there is the potential that this information might not be kept confidential (for instance by theft of study material).

4). Potential Risks of DXA scan: The total exposure resulting from these imaging studies is calculated to be approximately 0.135 mSv for three whole body DXA scans. This amount is less than you would receive from one year of natural exposure in the San Diego area, which is approximately 1.6 mSv. Cumulative exposure from radiation may increase your risk of developing certain types of cancer in the future.

15. RISK MANAGEMENT PROCEDURES AND ADEQUACY OF RESOURCES

Risks of psychological assessments: Participants will receive consistent support from the group leader throughout the study. Participants will be told that they are free to choose not to answer any questions that may cause them distress if they wish.

Risks of Treatment: Participants will receive consistent support from the group leader throughout the study. Participants will be told that they are free to choose not to answer any questions that may cause them distress if they wish. Participants will be told that they can stop the psychophysiological assessments at any time.

For any unidentified/unreported psychiatric concerns identified during assessments for this project, we will execute the following protocol:

1. Participant will be notified of concerns identified.
2. Participant will be given a list of referrals in the community. Dr. Boutelle is the training director for the UCSD Eating Disorder clinic, and will be able to refer patients directly to the clinic and to a number of providers in the community. In terms of other psychiatric issues previously unidentified, we will refer to providers in the UCSD Psychiatry department, or to community providers.
3. If significant concern is warranted (participant reports suicidal ideation, significant binging and purging), participants will be immediately assessed by the clinical staff present at the meeting regarding severity and an appropriate psychiatric referral will be made. Participants experiencing significant psychological distress or discomfort will be discontinued from the protocol and referred for counseling with their consent. Dr. Boutelle will call the participant the following week to determine whether they have followed through on referrals. If a participant has not followed through, they will be encouraged and will be offered help with following through (offer to make calls for the participant) if they choose to accept the assistance.

Potential risk of loss of confidentiality: The research team will make every effort to keep any information confidential. Any study material will be stored in locked cabinets in UCSD sponsored facilities. Furthermore, a unique identification number will be used for each person in data sets and spreadsheets that do not readily identify a name. The identifying name information containing material will be locked.

Risks of DXA scan: If the participant is especially concerned with radiation exposure, or has had numerous x-rays or imaging scans already, the participant should discuss this with the principal investigator for this study, Dr. Boutelle or Rhee, or their regular doctor. This information is included in the permission form and will be discussed with participant upon consenting for the study.

DSM: Because of this low risk status, the data and safety monitoring plan (DSMP) for this trial focuses on close monitoring by the principal investigator (PI) in conjunction with a safety officer, along with prompt reporting of excessive adverse events and any serious adverse events to the NIH and to the IRB at the University of California San

Diego. The safety officer for this trial will be Robert El-Kareh, MD, MS, MPH. Dr. El-Kareh is an internal medicine physician with an MPH in clinical effectiveness. Dr. El-Kareh has an in depth understanding of the types and severity of comorbidities and injuries associated with adult obesity. As Safety Officer, Dr. El-Kareh will review the reports sent by the study coordinator (at the frequency outlined below) and will use a checklist to determine whether there is any corrective action, trigger of an ad hoc review, or stopping rule violation that should be communicated to the study investigator, the University of California San Diego IRB, and the funding agency.

Safety reports will be sent to Dr. Strong (statistician), Dr. Boutelle (Principal Investigator), and the safety officer. The Project Coordinator will be responsible for assembling the data and producing these reports, as well as assuring that all parties obtain copies of these reports.

The frequency of data review for this study differs according to the type of data and can be summarized in the following table:

Data type	Frequency of review
Subject accrual (adherence to protocol regarding demographics, inclusion/exclusion)	At the end of each recruitment wave (monthly at the beginning of the study)
Adverse event rates (injuries)	Quarterly
Compliance to treatment	Quarterly
Stopping rules report regarding statistical power implications of drop outs and missing data	Yearly

Stopping rules

In this minimal risk intervention trial it is more likely that drop-outs or difficulty in recruiting adequate numbers of participants will require stopping the trial, than that of excess adverse events will occur and require stopping the trial. However, as outlined elsewhere, we will monitor injury rates in all participants and the safety officer, together with the study investigators, will alert the IRB and the NIH if a larger than reasonably expected injury rate should occur in the treatment group.

16. PRIVACY AND CONFIDENTIALITY CONSIDERATIONS INCLUDING DATA ACCESS AND MANAGEMENT

We will implement the following security plan to promote security of the data and privacy of the participants.

- Data collection will be completed with an emphasis upon maintenance of confidentiality. We plan to extract data from questionnaires and in-study behavioral measures. Only Dr. Boutelle /her research assistants, each of whom has mental health clinical training and UCSD clearance will have access to any personal health information collected. We will assign participants a study identification number unrelated to identifying information. The study ID number will be used by participants on their questionnaires and data collection forms. The only materials containing subject identifying information will be the consent and HIPAA forms. We will create a master list linking the de-identified study identification number to the participant's record. The record in the master list will be identified by a randomized participant number provided by our statistician. The master list will be maintained by Dr. Boutelle in her laboratory. There will be only one password protected electronic version of this file. Access to the master list will be limited to the P.I.s and their designees, all of whom will have completed UCSD IRB training requirements. At the earliest opportunity and no later than 36 months following data analysis, the master list (i.e., the only source that links the study identification numbers to the individuals) will be destroyed by Dr. Boutelle.

- We will create and maintain a separate password-protected electronic study database containing the de-

identified study identification number and study data specified above. The study database will be maintained on the UCSD secure network accessible only to UCSD-secured workstations. Individually identifiable health information (IIHI), as defined by Health Insurance Portability and Accountability Act of 1996 (HIPAA, Title II) will not be included in the study database. Specifically, we will exclude the 18 elements outlined by the Privacy Rule of HIPPA (section 164.514) and "safe harbor" definition to achieve de-identification of the study database. Access to the study database will also be limited to the P.I.s and their IRB trained research assistants.

Use of the study data will be limited to the proposed study. The IIHI and de-identified data will not be re-used and/or disclosed for purposes other than those outlined in this proposal. Further, we will not share the study data with other investigators, collaborators, and/or sponsors. Therefore, we will not transfer and/or transport the study database.

17. POTENTIAL BENEFITS

The most important potential benefit is the reduction of binge eating, which may translate into a number of favorable correlated outcomes, including improved weight loss, weight loss maintenance, and reductions in psychological distress.

18. RISK/BENEFIT RATIO

There is a relatively low risk to participants and the potential to benefit from a reduction in binge eating.

19. EXPENSE TO PARTICIPANT

There is no expense to participants for participating in this study. There is no cost to participate.

20. COMPENSATION FOR PARTICIPATION

All participants will receive incentives in the form of gift cards at the following levels for time and effort: mid-treatment = $25, post-treatment = $50, mid-follow-up = $25, and follow-up = $100.

21. PRIVILEGES/CERTIFICATIONS/LICENSES AND RESEARCH TEAM RESPONSIBILITIES

Kerri Boutelle, Ph.D (PI) is a professor in residence in the UCSD department of psychiatry and a licensed clinical psychologist. Her research specializes in the study of obesity and eating disorder behaviors. Dr. Boutelle is also a key developer of the food cue regulation treatment for binge eating being tested in this proposed study and has unique expertise in the application of this treatment.

David Strong, Ph.D (Co-I) is an associate professor in the department of family medicine and a licensed clinical psychologist. He has extensive experience managing data and conducting data analyses. His responsibilities include managing the databases and conducting data analyses for this project.

Cheryl Rock, Ph.D. (Co-I) is a Professor in the department of family medicine. She is responsible for overseeing the BWL curriculum and nutritional intervention materials and assisting in the supervision of BWL interventionists.

Bess Marcus, Ph.D. (Co-I) is a professor in the department of family medicine. She will be responsible for overseeing the physical activity recommendations and relevant curriculum as well as helping to advise recruitment and retention procedures.

June Liang Ph.D. (other) is the scientific director of the CHEAR lab. She will be responsible for training and supervising and certifying the assessors. She may assist in conducting assessments or intervention as well.

Dawn Eichen Ph.D. is a postdoctoral fellow. She will help train interventionists as well as assist with project coordination, assessment and intervention.

Teresa Monreal Ph.D. is a postdoctoral fellow. She will assist with project management as well as assessment.

Shari Neul is a licensed clinical psychologist. She will supervise the interventionist in the study and will help develop and manage all the treatment of participants for this study.

Martina Cotton is the study Project Coordinator. She will oversee the budgetary and administrative tasks management of the study, including the budget, hiring personnel, personnel issues, equipment management, NIH compliance and implementation of refinements to the protocols.

Monica Montoya is the study recruitment coordinator. She will coordinate all the recruitment and maintenance of the cohort for the study.

Ashley Ryan is the study Staff Research Associate. She will be responsible for subject recruitment, coordination of assessments, subject retention, data collection and entry, and monitoring compliance.

Adrienne Desens is the study data manager. She will be responsible for evaluating all data entry accuracy, merging of data files, creation of scales, and evaluating initial frequencies and means.

The following individuals are part of the CHEAR Assessment staff. They include graduate students and recent graduate program graduates who conduct clinical assessments and can serve as group leaders or co-leaders. They may also introduce the study and obtain consent. Everyone has CITI training:
Maritza Contreras-Rivera
Paulina Huh
Natalie Jones
Brittany Matheson
Lindsey McCutcheon
Melissa Mello
Zoe Mestre
Alexandra Ruhl
Sarah Speers

The following individuals are Research Assistants at CHEAR. These include undergraduate research assistants who help oversee the assessments and introduce the studies to the participants and may obtain consent. They also may help assist with group material preparation and obtaining heights/weights. Everyone has CITI training.
Research assistants:
Natalie Alamo
Jannet Chen
Gwendolyn Cheng
Emily Chung
Cyrielle Hacher
Pardeep Kaur
Joanne Kwak
Louis Langi
Francesca Lazzaro
Whitney Liu
Mishel Navarrete

Wagner Peng
Adriana Rodriguez
Sarah To

22. BIBLIOGRAPHY

1. Nederkoorn C, Smulders FTY, Jansen A. Cephalic phase responses, craving and food intake in normal subjects. Appetite. 2000;35(1):45-55.

2. Nederkoorn C, Jansen A. Food cue reactivity and regulation of food intake. Psychophysiology. 1999;36:S84-S84.

3. Castellanos EH, Charboneau E, Dietrich MS, et al. Obese adults have visual attention bias for food cue images: evidence for altered reward system function. Int J Obes. 2009;33(9):1063-1073.

4. Ferriday D, Brunstrom JM. 'I just can't help myself': Effects of food-cue exposure in overweight and lean individuals. Int J Obesity. 2011;35(1):142-149.

5. Nederkoorn C, Smulders FTY, Jansen A. Recording of swallowing events using electromyography as a non-invasive measurement of salivation. Appetite. 1999;33(3):361-369.

6. Boutelle KN, Norman GJ, Rock CL, Rhee KE, Crow SJ. Guided self-help for the treatment of pediatric obesity. Pediatrics. 2013;131(5):e1435-1442.

7. Appelhans BM, Luecken LJ. Heart rate variability as an index of regulated emotional responding. Review of General Psychology. 2006;10(3):229-240.

8. Camm AJ, Malik M, Bigger JT, et al. Heart rate variability. Standards of measurement, physiological interpretation, and clinical use. European Heart Journal. 1996;17(3):354-381.

9. Nederkoorn C, Coelho JS, Guerrieri R, Houben K, Jansen A. Specificity of the failure to inhibit responses in overweight children. Appetite. 2012;59(2):409-413.

10. Epstein LH, Carr KA, Lin H, Fletcher KD. Food reinforcement, energy intake, and macronutrient choice. Am J Clin Nutr. 2011;94(1):12-18.

11. Barratt W. The Barratt Simplified Measure of Social Status (BSMSS) measuring SES. 2006. Indiana State University; 2006.

12. Cooper A, Fairburn CG. The Eating Disorder Examination: A semi-structured interview for the assessment of the specific psychopathology of eating disorders. International Journal of Eating Disorders. 1987;6:1-8.

13. Gormally J, Black S, Daston S, Rardin D. The Assessment of Binge Eating Severity among Obese Persons. Addictive Behaviors. 1982;7(1):47-55.

14. Derogatis LR, Melisaratos N. The Brief Symptom Inventory: An introductory report. Psychol Med. 1983;13(3):595-605.

15. Brown, R.L., and Rounds, L.A. (1995). Conjoint screening questionnaires for alcohol and drug abuse. Wisconsin

Medical Journal 94:135-140.

16. Norman, S. B., Cissell, S. H., Means-Christensen, A. J. & Stein, M. B. (2006). Development and validation of an Overall Anxiety Severity And Impairment Scale (OASIS). DEPRESSION AND ANXIETY 23:245–249.

17. Kroenke K, Spitzer RL, Williams JBW. The PHQ-9: Validity of a brief depression severity measure. J Gen Intern Med 2001;16:606-613Lecrubier, Y., Sheehan, D. V., Weiller, E., Amorim, P., Bonora, I., Harnett Sheehan, K., ... & Dunbar, G. C. (1997). The Mini International Neuropsychiatric Interview (MINI). A short diagnostic structured interview: reliability and validity according to the CIDI. European psychiatry, 12(5), 224-231.

18. Shomaker, L. B., Tanofsky-Kraff, M., Mooreville, M., Reina, S. A., Courville, A. B., Field, S. E., . . . Yanovski, J. A. (2013). Links of adolescent- and parent-reported eating in the absence of hunger with observed eating in the absence of hunger. Obesity, 21(6), 1243-1250. doi:http://dx.doi.org/10.1002/oby.20218

19. Lecrubier, Y., Sheehan, D. V., Weiller, E., Amorim, P., Bonora, I., Sheehan, K. H., ... & Dunbar, G. C. (1997). The Mini International Neuropsychiatric Interview (MINI). A short diagnostic structured interview: reliability and validity according to the CIDI. European psychiatry, 12(5), 224-231.

20. Tylka TL. Development and psychometric evaluation of a measure of intuitive eating. Journal of Counseling Psychology. 2006;53(2):226-240.

21. Lowe MR, Butryn ML, Didie ER, et al. The Power of Food Scale. A new measure of the psychological influence of the food environment. Appetite. 2009;53(1):114-118.

22. Resnicow K, Odom E, Wang T, et al. Validation of three food frequency questionnaires and 24-hour recalls with serum carotenoid levels in a sample of African-American adults. American Journal of Epidemiology. 2000;152(11):1072-1080.

23. Olendzki B, Hurley TG, Hebert JR, et al. Comparing food intake using the Dietary Risk Assessment with multiple 24-hour dietary recalls and the 7-Day Dietary Recall. Journal of the American Dietetic Association. 1999;99(11):1433-1439.

24. Howat PM, Mohan R, Champagne C, Monlezun C, Wozniak P, Bray GA. Validity and reliability of reported dietary intake data. Journal of the American Dietetic Association. 1994;94(2):169-173.

25. Galasso R, Panico S, Celentano E, Del Pezzo M. Relative validity of multiple telephone versus face-to-face 24-hour dietary recalls. Ann Epidemiol. 1994;4(4):332-336.

26. Stunkard AJ, Messick S. The three-factor eating questionnaire to measure dietary restraint, disinhibition and hunger. Journal of Psychosomatic Research 1985;29(1):71-83.

27. Epstein LH, Leddy JJ, Temple J, Faith M. Food reinforcement: A multilevel analysis. Psychological Bulletin. 2007;133:884–906.

28. Epstein LH, Wright SM, Paluch RA, Leddy J, Hawk LW, Jr, Jaroni JL, et al. Food hedonics and reinforcement as

determinants of laboratory intake in smokers. Physiology and Behavior. 2004;81:511–517.

29. Godin, G., & Shephard, R.J. (1985). A simple method to assess exercise behavior in the community. Canadian Journal of Applied Sport Sciences, 10(3):141-6.

23. FUNDING SUPPORT FOR THIS STUDY

Funding for this study will be provided by the National Institute of Health, Diabetes, Digestive and Kidney Diseases, Agency award number 1 R01 DK103554.

24. BIOLOGICAL MATERIALS TRANSFER AGREEMENT

Not applicable. No biological materials will be collected or transferred.

25. INVESTIGATIONAL DRUG FACT SHEET AND IND/IDE HOLDER

Not applicable. No drugs will be given or investigated in this study.

26. IMPACT ON STAFF

Not applicable. This study does not involve the nursing staff from UCSD and/or RCHSD staff

27. CONFLICT OF INTEREST

There are no conflicts of interest.

28. SUPPLEMENTAL INSTRUCTIONS FOR CANCER-RELATED STUDIES

Not applicable.

29. OTHER APPROVALS/REGULATED MATERIALS

None.

30. PROCEDURES FOR SURROGATE CONSENT AND/OR DECISIONAL CAPACITY ASSESSMENT

Not applicable. Surrogate consent will not be used for this study.
