# Supplementary material for: Effect of a Novel Intervention Targeting Appetitive Traits on Body Mass Index Among Adults With Overweight or Obesity: A Randomized Clinical Trial
Source: JAMA Netw Open. 2022 May 18;5(5):e2212354. doi: 10.1001/jamanetworkopen.2022.12354 (PMC9118075; doi:10.1001/jamanetworkopen.2022.12354)
Supplement: Supplement 2. — eTable. Primary Outcome Analysis of Relationship Between Treatment Allocation and Percent Body Fat Measured With Dual-Energy X-Ray Absorptiometry at Posttreatment (Month 12) and 12-Month Follow-Up (Month 24) Assessments [file jamanetwopen-e2212354-s002.pdf]

Supplemental Online Content

Boutelle KN, Eichen DM, Peterson CB, et al. Effect of a novel intervention targeting appetitive traits on body mass index among adults with overweight or obesity: a randomized clinical trial. *JAMA Netw Open*. 2022;5(5):e2212354. doi:10.1001/jamanetworkopen.2022.12354

eTable. Primary Outcome Analysis of Relationship Between Treatment Allocation and Percent Body Fat Measured With Dual-Energy X-Ray Absorptiometry at Posttreatment (Month 12) and 12-Month Follow-Up (Month 24) Assessments

This supplemental material has been provided by the authors to give readers additional information about their work.

© 2022 Boutelle KN et al. *JAMA Network Open.*

eTable. Primary Outcome Analysis of Relationship Between Treatment Allocation and Percent Body Fat Measured With Dual-Energy X-Ray Absorptiometry at Posttreatment (Month 12) and 12-Month Follow-Up (Month 24) Assessments Note: Body composition measurements available for sub-sample completing the DXA procedure (ROC n = 32; BWL n = 33; ROC+ n = 33; AC n = 28).

	**Body Fat%**	
	Comparison with AC	Comparison with BWL
	Est (2.5%,97.5%)	Est (2.5%,97.5%)
Difference at End of Treatment		
AC	Ref	1.56 (-0.18, 3.29)
ROC	0.19 (-1.58,1.94)	1.74 (0.01, 3.47)
ROC+	-1.58* (-3.71, -0.22)	-0.41 (-2.04,1.23)
BWL	-1.55 (-3.29, 0.19)	Ref
Difference in Rate of Change from Post-Treatment to Final Follow-up		
AC	Ref	-1.35 (-2.73, 0.03)
ROC	-0.89 (-2.34,0.55)	2.24* (-3.59, -0.89)
ROC+	0.34 (-1.07,1.74)	-1.01 (-2.32, 0.29)
BWL	1.35 (-0.03, 2.73)	Ref

Note: AC = Active Comparator; ROC = Regulation of Cues; ROC+= Regulation of Cues + Behavioral Weight Loss; BWL = Behavioral Weight Loss. All models include covariates for age, sex, race-ethnicity, physical activity, loss of control eating and baseline percent body fat. Estimates (Est) and credible intervals (2.5% to 97.5% intervals reflect a 95% probability of where the true estimate would lie, given the current study data) are estimated from joint imputation models. * = Bayesian tail probability p<0.05

© 2022 Boutelle KN et al. *JAMA Network Open.*
