# Supplementary material for: Effect of a Novel Intervention Targeting Appetitive Traits on Body Mass Index Among Adults With Overweight or Obesity: A Randomized Clinical Trial
Source: JAMA Netw Open. 2022 May 18;5(5):e2212354. doi: 10.1001/jamanetworkopen.2022.12354 (PMC9118075; doi:10.1001/jamanetworkopen.2022.12354)
Supplement: Supplement 3. — Data Sharing Statement [file jamanetwopen-e2212354-s00.pdf]

Data Sharing Statement

Boutelle. Effect of a Novel Intervention Targeting Appetitive Traits on Body Mass Index Among Adults With Overweight or Obesity. *JAMA Netw Open*. Published May 18, 2022. doi:10.1001/jamanetworkopen.2022.12354

Data
Data available: Yes
Data types: Deidentified participant data
How to access data: Kboutelle@health.ucsd.edu
When available: With publication

Supporting Documents
Document types: None

Additional Information
Who can access the data: Researchers whose proposed use of the data has been approved
Types of analyses: Secondary analyses approved by the research team
Mechanisms of data availability: After approval of a proposal
